# Conditioned medium obtained from human amniotic membrane-derived mesenchymal stem cell attenuates heart failure injury in rats

**DOI:** 10.22038/ijbms.2019.36617.8722

**Published:** 2019-11

**Authors:** Solmaz Nasseri Maleki, Nahid Aboutaleb, Donya Nazarinia, Sara Allahverdi Beik, Asadollah Qolamian, Maliheh Nobakht

**Affiliations:** 1 Physiology Research Center and Department of Physiology, Faculty of Medicine, Iran University of Medical Sciences, Tehran, Iran; 2 Department of Physiology, Faculty of Medicine, Iran University of Medical Sciences, Tehran, Iran; 3 Department of Histology and Neuroscience, Anti-microbial Resistance Research Center, School of Medicine, Iran University of Medical Sciences, Tehran, Iran

**Keywords:** Adipose tissue-derived-mesenchymal stem cells, Apoptosis, Conditioned medium, Fibrosis, Heart failure

## Abstract

**Objective(s):**

Heart failure (HF) is one of the leading causes of death worldwide. Due to beneficial effects of stem cells, paracrine secretion of them has recently been used by researchers. The purpose of this study was to investigate the effects of intravenous injection (IV) of conditioned medium (CM) of human amniotic membrane-derived mesenchymal stem cell (MSC-CM) on HF.

**Materials and Methods:**

Male Wistar rats (n=35, 180 g) were randomly divided into five groups: sham, HF, HF+MSC-CM, HF+culture medium and HF+phosphate-buffered saline (PBS). To induce HF, isoproterenol (170 mg/kg/d) was injected subcutaneously for 4 consecutive days. After 28 days, induction of HF was evaluated by echocardiography. A day after echocardiography, 50 μg culture medium/5 ml PBS in HF+culture medium group, 50 μg MSC-CM/5 ml PBS in HF+MSC-CM group and 5 ml PBS in HF+PBS group were injected two times for 4 successive days. The echocardiography was performed 4 weeks after the last injection of isoproterenol. To evaluate the fibrosis, morphology, and cardiac function, Trichrome Masson’s staining, Hematoxylin and Eosin staining and echocardiography were performed, respectively.

**Results:**

CM significantly increased fractional shortening and ejection fraction, and also significantly decreased apoptotic nuclear condensation. Moreover, significant decreased level of fibrosis and increased level of angiogenesis was observed in the treatment group (*P<*0.05).

**Conclusion:**

Our results indicated that IV injection of CM has therapeutic effects on HF by reducing fibrosis and preventing the progression of failure due to its paracrine effects.

## Introduction

Heart failure (HF) is one of the most important causes of morbidity and mortality that is considered as a major challenge around the world because it causes 10-13% of global death (1). HF is a major threat to public health owing to lack of signs in early stages and awareness of patients in late-stage (2, 3). In recent years, researchers have used many novel options to treat degenerative disorders and other diseases (4-7). Stem cell-based therapy has attracted much attention from scientific community as an appropriate therapeutic approach (8, 9). For instance, Teerlink and colleagues reported that intramyocardial administration of cardiopoietic mesenchymal stem cells (MSCs) exhibited beneficial effects on left ventricular (LV) reverse remodeling as a pivotal marker of improved outcomes in patients with advanced HF (10). Likewise, Bartolucci and colleagues reported that intravenous administration of umbilical cord MSCs in patients with HF resulted in improvements in LV function, functional status, and subsequently quality of life (11). Several mechanisms of stem cells in regeneration of cardiac tissue after HF have been detected. These include differentiation into cardiomyocytes and myocardial regeneration (12), differentiation into vascular smooth muscle, endothelial cells and subsequently angiogenesis (13), and secretion of paracrine factors with protective effects by these cells (14). Although using stem cell-based therapies in rehabilitation of tissue in degenerative disorders has garnered tremendous attention in recent years, their clinical applications have been challenged by concerns regarding their serious side effects such as tumorigenesis (particularly in the case of embryonic stem cells) (15), ethical considerations, undesired differentiation into other body organs, evoking of immune response, and sometimes painful procedures (16, 17). On the other hand, although sometimes myoblasts have well-differentiated into ventricular cardiomyocytes, owing to heart arrhythmias they were not good candidates for regeneration of damaged tissues. However, in the treatment of cardiovascular diseases using stem cells, it was believed that these cells turn into cardiomyocytes and vascular endothelial cells. Other results have shown that the differentiation of these cells into useful cells is still debated (18). Studies have shown that a large proportion of intravenously injected stem cells into rodents are trapped in the lungs of the animals. Likewise, migration and poor survival of stem cells in the site of injury are other serious problems (19). Instead, the release of anti-inflammatory factors can cause therapeutic effects (20). 

Currently, it is well-documented that MSCs can secrete a number of neurotrophic paracrine and immune modulatory factors that contribute to repair of the damaged tissues (21, 22). Indeed, although it was primarily hypothesized that the action mechanism of MSCs is associated with cell replacement, recently focus has changed to their paracrine actions (23).

 Researchers have observed that conditioned medium (CM) secreted by stem cells can exert protective effects through stimulation of angiogenesis and improvement of tissue function (24). CM secreted by stem cells possess a large number of proangiogenic, antiapoptotic, and growth factors including hepatocyte growth factor (HGF), angiogenin, interleukin 8 (IL-8), stromal cell-derived factor 1 (SDF-1), insulin-like growth factor-1 (IGF-1), vascular endothelial growth factor (VEGF), and exosomes (25). These factors can contribute to stimulation of angiogenesis, improvement of endothelial tube formation, recovery of cardiac function and improvement of cell survival through alterations in expression profile of many genes and proteins (26, 27). On the basis of the aforementioned studies, we investigated cardioprotective effects of MSC-CM against HF. Here, we indicated that intravenously administrated CM markedly restored heart function and reduced fibrosis in a rat model of HF.

## Materials and Methods

***Animals and chemicals***

Male Wistar rats (35 rats, body weight: 180 g) were randomly divided into five groups. The animals were housed in an animal room at 25±3 ^°^C under a 12 hr dark and 12 hr light cycle with free access to standard pellet chow and water *ad libitum*. The procedures were performed in accordance with the Guide for the Care and Use of Laboratory Animals published by the US National Institutes of Health (NIH Publication No 85-23, revised 1996) and approved by Animal Ethical Committee of Iran University of Medical Sciences (reference number: IR.IUMS.REC 1395.9411344002). All chemicals and reagents were obtained from commercial sources in the highest quality available. 

***Experimental design***

The animals were divided into five groups: a sham group (sham; n=7), a group of rats were only subjected to heart failure (HF; n=7), a group of rats were subjected to heart failure and treated with CM in 4 consecutive days (HF+MSC-CM; n=7), a group of rats were subjected to heart failure and received culture media in 4 consecutive days (HF+culture medium; n=7), and a group of animals were subjected to heart failure and received phosphate-buffered saline (PBS) in 4 consecutive days (HF+PBS; n=7). The echocardiography was performed on the first day (baseline), 28 days (for approving the induction of heart failure) and then 61 days later. After confirmation of HF by echocardiography on 28 days after the last injection of isoproterenol, 50 μg/5 ml PBS of MSC-CM and culture medium were intravenously administrated (two times per day) for 4 consecutive days in HF+MSC-CM and HF+culture medium groups, respectively. In the case of the group HF+PBS, only 5 ml PBS was intravenously administrated (two times per day) for 4 consecutive days. Ejection fraction (EF) less than 40% was considered as severe degrees of heart failure. At the end of experimental period, rat hearts were isolated under deep anesthesia and stored at -80 ^°^C prior to biochemical assessments. 

***Preparation of human amniotic membrane-derived mesenchymal cell***

Human amniotic membrane-derived mesenchymal cells were prepared from Iranian Blood Transfusion Organization. The best time for preparation of MSC-CM is after the third and before the fifth passage. 

***Identification*** ***of human MSC***

To analyze cultured MSCs, we performed fluorescence-activated cell sorting (FACS) as described in our previous study (28).

The cell surface markers such as CD 29, CD 45, CD 166, and CD105 were evaluated to ensure the acceptable purity of these cells. These antibodies are an indication of the cells in mesenchymal stage. Trypsinized cells (1×10^6^) were incubated with above antibodies at concentration of 5 ug/ml in PBS for 20 min under dark condition.

***Preparation of CM***

After the third passage, MSCs were cultured in flask 75. After washing with PBS, cells were incubated in MEM-α (minimum essential medium α modification). The CM was harvested through incubation of cells in a hypoxic incubator (94% N_2_, 5% CO_2_ and 1% O_2_) for 48 hr followed by centrifugation for 10 min at 1200 rpm and filtration with 0.22 µm filter. The resulting solution was CM without MSC. Prepared CM and culture media were lyophilized in vials. Lyophilized products were kept in freeze drying machine for 24 hr.

***Heart failure model***

On the basis of the aforementioned studies, excessive administration of catecholamines results in tremendous myocardium destruction and fibrosis (29). To induce the global heart failure, 170 mg/kg/d isoproterenol was solved in 0.5 ml saline and were injected subcutaneously once a day in four consecutive days. 

***Histological studies***

At the end of experimental period, the animals were sacrificed under deep anesthesia. Hearts were isolated and were fixed in 4% formaldehyde solution for 3 days. Then, paraffin blocks were prepared by tissue processor and sections (7 microns) were prepared by microtome.

In order to identify and assess the fibrosis level, Trichrome Masson’s staining was used. Hematoxylin and Eosin staining was also used to evaluate the nuclei and texture morphology**.**

***Statistical analysis***

All values were expressed as mean±standard deviation (SD). Analysis of data was performed using Graph-Pad Prism-5 statistical software (La Jolla, CA). The data of parametric variables between different groups were analyzed using one-way ANOVA followed by Tukey *post hoc* test. Differences were considered statistically significant when *P*<0.05. 

**Figure 1:**
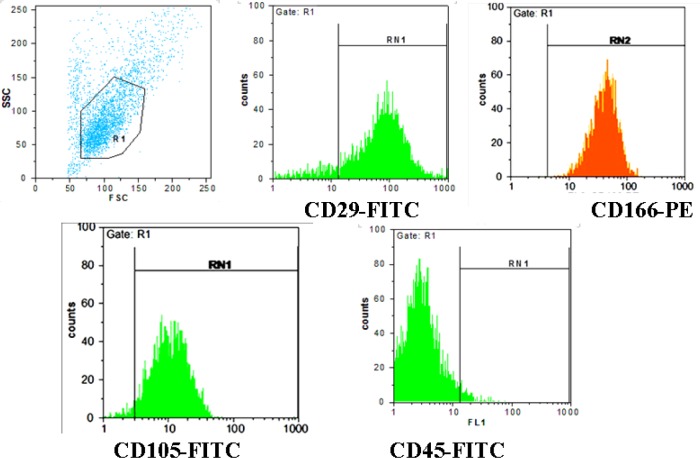
Evaluating the expression of surface markers of mesenchymal stromal cells (MSCs) and hematopoietic cells (CD29, CD105, CD45, CD166). Most cultured cells showed high expression of CD29, CD105, and CD166, indicating isolation of a highly purified MSC population. On the other hand, the majority of cultured cells were CD45 negative

**Figure 2:**
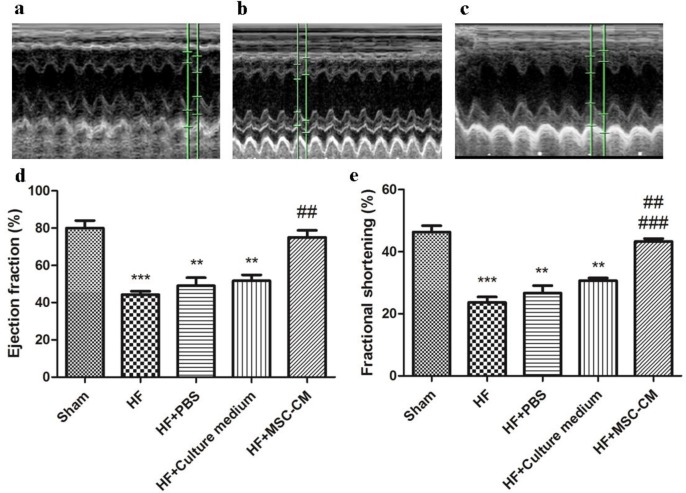
Administration of conditioned medium of human amniotic membrane-derived mesenchymal stem cell (MSC-CM; twice per day for 4 consecutive days after induction of heart failure (HF)) significantly restored cardiac function through improvement of fractional shortening (FS) and ejection fraction (EF). a) Sham group showed a normal FS and EF. b) HF group showed significant decreases in FS and EF. c) MSC-CM group indicated significant restoration of FS and EF compared to HF, HF+phosphate-buffered saline (PBS), and HF+CM. Quantitative analysis of d) EF (***P<0.001, ** *P<*0. 01 compared to sham; ##*P<*0. 01 compared to HF; HF+PBS, and HF+culture medium), and e) FS (***P<0.001, ** *P<*0. 01 compared to sham; ##*P<*0. 01 and ### *P<*0. 001 compared to HF; HF+PBS, and HF+culture medium)

**Figure 3:**
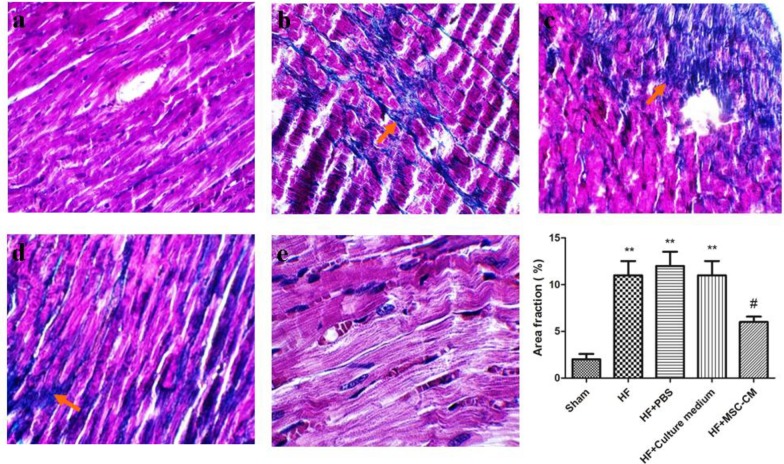
Administration of conditioned medium of human amniotic membrane-derived mesenchymal stem cell (MSC-CM; twice per day for 4 consecutive days after induction of heart failure (HF)) significantly reduced cardiac fibrosis (blue parts in images show collagen synthesis and deposition). a) Sham group did not show any sign of fibrosis. b) An increased collagen synthesis and deposition was found in HF. c and d) administration of phosphate-buffered saline (PBS) and culture medium did not contribute to reduction of fibrosis. e) A reduced level of fibrosis was observed after administration of MSC-CM twice per day for 4 consecutive days (** *P<*0. 01 compared to sham; # *P<*0. 05 compared to HF; HF+PBS, and HF+culture medium, red arrows show collagen synthesis and deposition)

**Figure 4:**
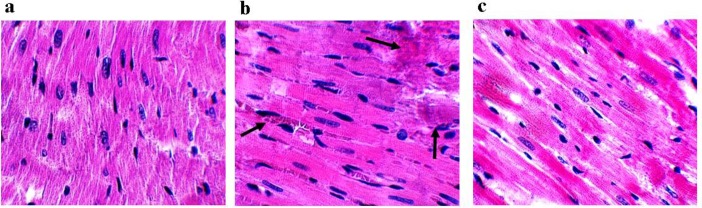
Conditioned medium of human amniotic membrane-derived mesenchymal stem cell (MSC-CM) post- treatment markedly reduced tissue damage (black arrows show tissue damage). a) The cytoarchitecture of myocardial wall and morphology were normal in sham group. b) Heart failure (HF) demonstrated an abnormal cytoarchitecture of the myocardial wall and chromatin condensation in apoptotic nucleus. c) An improved cytoarchitecture of the myocardial wall and reduced level of chromatin condensation was found in rats treated with MSC-CM

## Results

***Characterization of MSC by flow cytometry***

The results of flowcytometric analysis showed that the expression of specific markers of MSC including CD29, CD105, and CD166 were high in human amniotic membrane isolated cells (Figure 1). These results confirmed the isolation of MSC and removal of hematopoietic cells during the isolation of MSC. As shown in Figure 1, CD markers 29, 105 and 166 (surface markers for MSCs) are located in positive area that is an indication for expression of these proteins in the cells for approval of MSCs. As shown in Figure 1, CD marker 45 (surface markers for hematopoietic cells) is located significantly in negative areas, which is an indication for lack of expression of this marker (or little expression) in cultured cells; it is an acceptable indicator for non-hematopoietic cell in cultured cells. These data confirm isolation of a highly purified MSC population.

***Heart function***

To evaluate cardiac function in different groups, we performed echocardiography (Figure 2 a-c). Our results showed that the EF was significantly decreased in HF compared to sham, suggesting induction of HF in rats subjected to isoproterenol for 4 consecutive days (*P*<0.05). HF+MSC-CM group revealed a significant increase in EF compared to HF group. Quantitative analysis showed that the average EF in HF group was 44% that increased to 75% in the HF+MSC-CM group (Figure 2 d and e). There were no significant differences between HF+culture medium, HF+PBS, and HF. The fractional shortening (FS) was markedly decreased in HF relative to sham. MSC-CM administration twice per day for 4 consecutive days markedly restored HF. No significant differences were observed between HF+culture medium, HF+PBS, and HF.

***Evaluation of fibrosis***

To obtain greater insights into protective effects of MSC-CM against HF, we evaluated collagen synthesis and deposition using Trichrome Masson’s staining. Sham group did not show any fibrosis (Figure 3a)**. **Trichrome Masson’s staining demonstrated that induction of HF markedly resulted in irreversible loss of a large number of cardiomyocytes and extension of fibrosis (Figure 3b; blue color). Administration of MSC-CM markedly blunted the extension of fibrosis (Figure 3e). A significant reduced fibrosis was not observed in HF+culture medium and HF+PBS relative to HF (Figure 3c and 3d). 

***Assessment of tissue damage***

To approve the above results about protective effects of MSC-CM against HF, we evaluated tissue damage using H&E staining. Sham heart tissues demonstrated normal morphology without any abnormal cytoarchitecture of the myocardial wall and chromatin condensation in apoptotic nucleus (Figure 4a). 

Severe damages such as abnormal cytoarchitecture of the myocardial wall and apoptotic nuclear condensation were observed in HF group (Figure 4b). Administration of MSC-CM remarkably preserved structure and morphology of cardiomyocytes and reduced the level of chromatin condensation (Figure 4c). 

## Discussion

Discovery of new therapeutic options for degenerative disorders is an utmost issue owing to growing list of these diseases in recent years (30-34). Stem cell-based therapies have attracted much attention in scientific community because they lead to regeneration of damaged tissue (35). Proliferation and differentiation of stem cells in the site of injury can contribute to improvement of tissue function and the lifespan extension in patients. Although stem cell-based therapies have many advantages, some drawbacks might limit their clinical applications. Some studies have reported lack of differentiation of these cells or low efficiency or lack of coordination with the desired tissue. For example, it has been reported that after stem cell transplantation in heart, these cells have differentiated into cardiomyocytes, but they have not shown sufficient improvement as they could not coordinate with the original heart rate and contraction. Other drawbacks of stem cell-based therapies that may limit their applications in routine clinical practice are rapid migration to other tissue organs and poor survival. For instance, previous studies have shown that the number of donor cells in the heart rapidly decreased from 80% to 34% in early hours (19). Many researchers have examined various ways to solve this problem (36). Moreover, aberrant migration or distribution of stem cells to other organs such as liver, spleen, and lung might result in tissue dysfunction or increase risk of tumorigenesis (37). Aside from pivotal role of stem cells in regeneration of injured tissues, some recent studies have shown that CM of these cells might have beneficial effects to improve tissue function (38). Our findings showed that the use of CM reduced the level of fibrosis. These results can be attributed mainly to presence of the therapeutic agents in paracrine secretion of these cells. In keeping with our results, it has been shown that CM secreted from these cells includes coding and non-coding RNA that can be translated into a specific protein or affect genes expression. It is well-documented that CM can enhance angiogenesis (39) (38-42). To gain greater insights into protective effects of CM, we examined heart function. Our results showed that CM administration markedly restored heart function through restoration of EF and FS. In consistent with our finding, a previous study showed that paracrine effects of MSCs play an important role in improvement of cardiac function after acute myocardial infarction (MI) (40). Likewise, another study showed that MSC-CM injection after MI contributed to restoration of cardiac function (41). It seems that factors secreted from MSCs have therapeutic effects on heart failure by reducing the injury level and preventing the progression of failure. Intravenous injection injection of CM can be used in patients for cardiac function improvement over time. Because MSCs have immunomodulatory effects and can be prepared easily from fetal unusable membrane and also have ethical considerations, it seems that using CM of these cells is very appropriate. It does not also require surgery and aggressive manipulation, so it can prevent problems and other side effects in patients.

## Conclusion

Collectively, our findings showed that CM of hMSCs might be a good candidate to reduce cardiac injury caused by HF. Although we demonstrated that CM of hMSCs restored cardiac function and reduced fibrosis in a rat model of HF, but more research is needed for clinical applications. 
